# Integrated malaria prevention in rural communities in Uganda: a qualitative feasibility study for a randomised controlled trial

**DOI:** 10.1186/s40814-021-00894-0

**Published:** 2021-08-10

**Authors:** David Musoke, Carol Namata, Rawlance Ndejjo, John C. Ssempebwa, Miph B. Musoke

**Affiliations:** 1 Department of Disease Control and Environmental Health, School of Public Health, College of Health Sciences, Makerere University, Kampala, Uganda; 2 Department of Applied Sciences, School of Sciences, Nkumba University, Entebbe, Uganda

**Keywords:** Malaria prevention, Feasibility study, Randomised controlled trial, Integrated approach, Integrated malaria prevention, Uganda

## Abstract

**Background:**

A randomised controlled trial (RCT) on integrated malaria prevention, which advocates the use of several malaria prevention methods holistically, has been proposed. However, before conducting an RCT, it is recommended that a feasibility study is carried out to provide information to support the main study, particularly for such a complex intervention. Therefore, a feasibility study for an RCT on integrated malaria prevention in Uganda was conducted.

**Methods:**

The qualitative study carried out in Wakiso District employed focus group discussions (FGDs) and key informant interviews (KIIs) to explore community willingness to participate in the RCT as well as assess stakeholder perspectives on the future study. The participants of the FGDs were community members, while the key informants were selected from malaria stakeholders including Ministry of Health officials, health practitioners, local leaders, district health team members, and community health workers (CHWs). Thematic analysis was employed with the support of NVivo.

**Results:**

A total of 12 FGDs and 19 KIIs were conducted. Five main themes emerged from the study: malaria prevention practices related to integrated malaria prevention; preferred malaria prevention methods in the integrated approach; potential challenges of integrated malaria prevention; perspectives on the proposed RCT; and sustainability of integrated malaria prevention. Despite a few methods being employed holistically in the community, insecticide-treated nets were the most widely used and preferred method for malaria prevention mainly because they were provided free by the government. The main challenges in the integrated approach were the high cost of some methods such as house screening, and concerns about the potential side effects of insecticide-based methods such as indoor residual spraying. Participants expressed high willingness to participate in the RCT to promote the use of multiple methods in their households and community. Involvement of CHWs during implementation was proposed as a sustainability strategy for the RCT interventions.

**Conclusion:**

There was high willingness to participate in the proposed RCT on integrated malaria prevention. However, high cost and perceived negative health effects from some methods were identified as potential challenges. The type of methods to be included as well as sustainability mechanisms needs to be considered during the design of the RCT.

## Key messages regarding feasibility

- Our previous project that promoted integrated malaria prevention in rural Uganda was well received by the community and showed promise in contributing to national malaria prevention efforts. To further explore the integrated approach, a randomised controlled trial (RCT) is needed to quantitatively measure the public health impact of the strategy in contributing to malaria prevention efforts. However, there were uncertainties regarding willingness of households to participate in the RCT, and any challenges that may be encountered.
- Although there was high willingness to participate in the proposed RCT on integrated malaria prevention, high cost and perceived negative health effects from some methods were identified as potential challenges.
- Careful consideration is needed whilst choosing the type and number of methods to be included in the RCT.

## Background

In 2019, it was estimated that there were 229 million malaria cases and 409,000 deaths globally [1]. Africa has the highest prevalence of malaria, and accounts for over 90% of all deaths from the disease with children under 5 years of age and pregnant women the most vulnerable [2]. These figures remain high despite global malaria estimated cases reducing by 20 million between 2010 and 2019 [1]. Estimates of the true burden of malaria have been difficult to obtain due to many malaria cases and deaths going unreported particularly in low- and middle-income countries (LMICs) [3]. This implies that the current malaria burden could be much higher than the estimates suggest. In Uganda, malaria is the leading cause of morbidity and mortality especially among children [4]. The disease accounts for approximately 30-50% of outpatient care in health facilities, 15-20% of health facility admissions, and up to 20% of all inpatient deaths in the country, 27% of which are among children under 5 years of age [5]. Among the highest malaria burden countries, Uganda ranks third globally on the estimated number of cases [1]. In addition to its impact on health, the burden of malaria on Uganda results in vast social and economic consequences including affecting societal engagements, contributing to high out of pocket health expenditure, and reducing productivity at school and work [6].

Globally, malaria prevention has mainly relied on mosquito vector control by using insecticide-treated nets (ITNs) and indoor residual spraying (IRS) [1, 3]. Indeed, malaria vector control strategies in Uganda have focused on the use of ITNs particularly long-lasting insecticidal nets (LLINs) and IRS. The use of LLINs in the country has significantly increased in recent years, and households that own at least one ITN are estimated at 83%, with 54% of households owning one ITN for every two household members [7]. The Ministry of Health (MOH) has provided free ITNs particularly to children under 5 years of age and pregnant women since 2006 [8, 9], most recently distributing 27.5 million LLINs in 133 districts across the country in 2020 [10]. These initiatives of increasing access to ITNs are aimed at preventing malaria deaths particularly among vulnerable groups in the country. Regarding IRS, a large regional variation in its coverage exists in Uganda due to intensive spraying interventions carried out in only 14 high burden districts in the northern and eastern parts of the country spearheaded by the MOH and non-governmental organisations (NGOs) [7]. Countrywide, only 10% of households had received IRS in the previous 12 months in 2018/19 compared to 74% in the IRS target districts [7]. Country-specific studies in Uganda have shown that the use of ITNs [11] and IRS [12] have reduced the occurrence of malaria in areas where they have been used. However, despite all these efforts, the burden of malaria in many parts of the country is still high [1, 5, 13].

Although global and national malaria vector control efforts have focused on ITNs and IRS, several other practices can be implemented at households to reduce mosquitoes which transmit the disease. These practices include the following: eliminating mosquito breeding sites notably stagnant water; installing screening in windows, vents and open eaves to prevent the entry of mosquitoes into houses; closing windows and doors early in the evenings to limit mosquito entry; spraying houses with insecticides to kill mosquitoes; use of body mosquito repellents and larviciding. These methods, despite being ignored in many malaria-endemic communities including in Uganda and elsewhere [14–16], are known to contribute to the reduction of mosquito populations, their entry into houses, and mosquito bites hence the occurrence of malaria [17–19]. Integrated malaria prevention is therefore being explored by the research team to reduce occurrence of the disease in Uganda [20–22]. This innovative approach advocates the use of several malaria prevention measures in a holistic manner within households and in communities. However, the use of all these methods is unlikely to be feasible, with several challenges anticipated such as the burden and time involved in implementing the many practices within households [21].

A previous project we conducted that promoted integrated malaria prevention in rural Uganda was well received by the community and showed promise in contributing to national malaria prevention efforts [20]. The benefits reported by households using the integrated approach, assessed qualitatively, included a reduction in the population of mosquitoes in houses, and occurrence of malaria [21]. In addition, a related study established a high willingness of the participants (68.6%) in using integrated malaria prevention if trained [20]. To further explore the integrated approach, a randomised controlled trial (RCT) is needed to quantitatively measure the public health impact of the strategy in contributing to malaria prevention efforts. However, before conducting an RCT, it is recommended that feasibility studies are carried out to provide information that is necessary to inform the main study [23] particularly for such a complex intervention [24]. Indeed, feasibility studies are crucial in providing information on implementation of the intervention including willingness of households to participate in the RCT, and any challenges that may be encountered. Therefore, a qualitative feasibility study for an RCT on integrated malaria prevention in rural communities in Uganda was conducted.

## Methods

### Study design and participants

The qualitative study carried out in rural Wakiso District, Uganda, used focus group discussions (FGDs) and key informant interviews (KIIs) to explore community willingness to participate in an RCT on integrated malaria prevention as well as assess stakeholder perspectives on the main study. Participants of the FGDs were identified and selected purposively by the local council 1 (village) chairpersons and community mobilisers in the area with guidance from the research team. These FGDs were conducted to generate information on households’ views of participating in the proposed RCT and related malaria prevention aspects. A total of 12 FGDs were conducted, with 4 each being held for adult men, adult women and youth, and each FGD had 8 or 9 participants. Separate FGDs for these groups were held so that views from each of them were obtained without being compromised by being in the presence of individuals from other categories. A total of 19 key informants were purposively selected based on their expertise and relevance to malaria prevention in Wakiso District and Uganda in general. These included 2 malaria researchers, 3 health practitioners from public health facilities, 3 local council 1 chairpersons, 3 community health workers (CHWs), 4 members of District Health Teams, 3 MOH National Malaria Control Division officers and 1 malaria technical personnel from an NGO. The KIIs were held to get views on the RCT and associated issues related to integrated malaria prevention. Data saturation was achieved on completion of the 12 FGDs and 19 KIIs conducted in the study.

### Study area and setting

The study was carried out in Kajjansi town council in Wakiso District, Uganda. Kajjansi town council has 11 parishes and consists of both peri-urban and rural communities with an estimated population of 94,238 (45,272 males and 47,966 females), 23,992 households, and an average household size of 3.8 [25]. The main economic activities carried out in the area include agriculture, small-scale trade, brick making and sand mining. Malaria is endemic in the area and is a leading cause of morbidity and mortality (as is the case in most parts of the country). The town council has 3 government health facilities of Kajjansi Health Centre IV, Nakawuka Health Centre III and Nsaggu Health Centre II, and several private facilities including clinics. In addition to offering curative services for malaria (and other diseases), many of these facilities are involved in malaria prevention initiatives such as distribution of LLINs to pregnant women during antenatal care. CHWs also exist in the area and are the first contact of the community with the health system. The CHWs offer several services related to malaria control including treatment of children under 5 years of age with malaria, pneumonia and diarrhoea under integrated community case management of childhood illnesses, household visiting for health improvement, referral of patients to health facilities and mobilisation of the community for health interventions such as distribution of ITNs. The town council has previously benefitted from MOH malaria prevention interventions notably receiving LLINs for the community.

### Sampling and data collection

Four predominantly rural parishes of Ssisa, Ngongolo, Nakawuka and Bulwanyi were purposively selected from the 11 parishes in Kajjansi town council for involvement in the study due to their higher malaria prevalence than urban settings. In addition, rural communities were predominantly selected for the feasibility study because these will be the target for the future main RCT. From these parishes, 10 villages of Kagulu, Namazzi, Kasuku, Mpumudde, Nakawuka A, Nakawuka B, Katwe, Lukose, Bulwanyi Central and Kaama II were purposively selected for the study. These villages were selected because of having a high prevalence of malaria, as well as many large pools of stagnating water resulting from brickmaking and sand mining in the community. At least one FGD was conducted in each of the villages, with two villages with the most pools of water hosting two discussions. These water pools are breeding places for mosquitoes hence playing a significant role in the transmission of malaria in the community. Some of the FGD participants were involved in brickmaking and sandmining, and were eager to embrace malaria prevention initiatives since they acknowledged also being affected by malaria. The FGDs were held in the respective villages of the participants at a central place such as the home of the local council 1 chairperson. KIIs were scheduled by telephone appointment, with the key informants suggesting the appropriate date, time and location for the interviews. Due to the COVID-19 pandemic, social distancing was maintained and masks were worn throughout the FGDs and KIIs. For some key informants, the interviews were conducted via telephone due to travel and meeting restrictions caused by the pandemic.

Semi-structured discussion and interview guides were used for the FGDs and KIIs respectively. The guides were piloted in a village in Wakiso District not involved in the study, and revisions made to the tools before actual data collection. The main revisions made were rephrasing some questions in the translated FGD guide to be easily understandable, and adding some more probes to the KII guide. The guides had questions related to community participation in the proposed RCT on integrated malaria prevention, as well as related aspects such as current malaria prevention practices and foreseeable challenges in the integrated approach. A description of the proposed future RCT was included in the guides to aid participants in responding to the questions. Both guides were developed in English and translated into *Luganda*, which is the local language mostly used in the study area. All data from the FGDs were collected in *Luganda* facilitated by a research assistant who also recorded all proceedings of the discussions. The research assistant was female and had a master’s degree in Public Health, with experience in qualitative research. The research assistant had no relationship with the participants, and none of her characteristics was expected to have any influence on the study. Most of the KIIs were conducted and recorded in English, with some interviews conducted in the local language such as with CHWs and local leaders. The KIIs were also conducted by the research assistant who was involved in the FGDs.

### Data management and analysis

All audio recordings of the FGDs and KIIs were transcribed verbatim and proof-read by the research assistant to ensure an accurate record of the proceedings. Once the transcripts were validated by the research assistant, those in *Luganda* were translated into English, and all were verified by the principal investigator. A thematic approach was employed in Nvivo12, a qualitative data analysis software, to analyse the data. During analysis, two researchers experienced in qualitative data analysis (DM and CN) commenced by developing a new project in Nvivo and imported the transcriptions. Thereafter, the researchers read through the imported texts of data, which was followed by the process of data coding. A codebook was developed through an iterative process that involved developing codes, their definitions and examples. The coding process involved assigning codes defined in the codebook to raw data [26]. The codes represented a link between parts of the text from the data and research questions. After coding the text, the researchers searched for connections between the various concepts, and identified as well as grouped conceptually similar codes that were identified as sub-themes. Using thematic analysis, the related sub-themes were then grouped together to form themes. The themes generated from the data analysis are presented as the major findings from the study. Quotations from the participants were selected and used during the presentations of the findings as appropriate. The findings are to be disseminated to the participants including through village meetings to be organised by the local leaders and community mobilisers.

### Ethical considerations

The study obtained ethical approval from Makerere University School of Public Health Higher Degrees, Research and Ethics Committee (866). Approval and registration of the study by the Uganda National Council for Science and Technology (HS999ES) was also done as required by local guidelines. Participation in the study was voluntary and participants provided written informed consent after explaining to them the proposed research, including the anticipated risks and potential benefits before they took part. Data were only accessed by the researchers and used solely for purposes of the study.

## Results

All individuals approached accepted to take part in the study for both the FGDs and KIIs. A total of 98 participants (49 males and 49 females) were involved in the 12 FGDs, in addition to the 19 key informants. The average duration of FGDs was 45 min whilst that of the KIIs was 30 min. Results from the study are presented under five main themes: malaria prevention practices related to integrated malaria prevention; preferred malaria prevention methods in the integrated approach; potential challenges of integrated malaria prevention; perspectives on the proposed RCT; and sustainability of integrated malaria prevention.

### Malaria prevention practices related to integrated malaria prevention

From the FGDs, only a few methods in the integrated approach were being used for malaria prevention in the community. For this reason, use of multiple malaria methods at households was noted to be minimal. It was established that the most widely used method of malaria prevention among community members was ITNs. Participants reported that the government gave them free ITNs as a key strategy to prevent the disease. However, some participants reported that although they were sleeping under ITNs, at times mosquitoes did bite them before going to bed. To avoid mosquitoes in houses, some participants reported that in addition to using ITNs, they endeavoured to clear overgrown vegetation that could potentially harbour the vectors, as well as drain stagnant water around their homes that could act as breeding grounds for mosquitoes.

"*What I normally do to prevent malaria is making sure that each of my children have their own treated mosquito net. As for the vegetation near my house, I fight to see that it is short, and I also drain all the stagnant water in the trenches."* Female participant 4, FGD 1

Some community members in the FGDs reported that they used mosquito coils at night before going to bed since they repel mosquitoes. However, some participants felt that mosquito coils could have harmful health effects and instead burnt dried cow dung which produced smoke to chase mosquitoes from households.

"*I sometimes use mosquito coils while in the sitting room where I burn them to drive away mosquitoes. This has helped me as a means of tackling mosquitoes before going to sleep.* Youth participant 4, FGD 11

It was also established from the FGDs that some community members used plants in the prevention of malaria. These participants planted neem trees and lemongrass in their compounds which they reported to have a mosquito-repellent odour preventing mosquitoes from getting to their houses. Other participants reported closing windows and doors on their houses early at sunset to prevent mosquitoes from entering.

"*We also plant trees for instance lemongrass in the compound to repel mosquitoes. This is a practice we used to do since I was young, particularly planting neem trees, and we would not get any mosquitoes."* Female participant 1, FGD 4

### Preferred malaria prevention methods in the integrated approach

From the FGDs, many of the participants preferred ITNs as they were provided free of charge by the government hence they did not spend money to buy them. In addition, ITNs were preferred as they were noted to be easy to maintain hygienically since they only involved washing, and acted as a barrier between the individuals and mosquitoes.

"*First of all, the ITNs are provided to us freely by the government, and it is so easy to keep these ITNs clean so that’s why they are easier to use in our households than any other method which would involve the use of money that we do not have*". Male participant 3, FGD 5

Additionally, some participants including local key informants preferred to instal screens in windows and vents compared to other methods like the use of ITNs. Since some participants complained about skin irritation when using ITNs, installing screens on houses was seen as a better option for these individuals as they said it had no side effects. In addition, some participants preferred house screening as they noted it was a long-lasting method of preventing malaria.

"*I think installing screening in windows and vents would be a better method. With this method, those who get skin rashes once they sleep under mosquito nets can sleep peacefully because they would have prevented mosquitoes from entering into their houses by installing screens so I think that it would be a better method to use."* Local leader, Lukose village

Some malaria prevention methods were preferred because of being simple and relatively easy to implement. For instance, some FGD participants preferred closing windows and doors at sunset to prevent the entry of mosquitoes into their houses or conducting practices such as clearing overgrown vegetation in the compound.

### Potential challenges of integrated malaria prevention

The main challenge that participants identified in the integrated approach was the high cost of some malaria prevention methods. It was established that it would be difficult to use malaria prevention methods that involved the use of money, such as mosquito coils, body mosquito repellents, installing screens in windows and vents, larviciding, insecticide spraying and IRS. In addition, some community members mentioned that the installation of screens in windows and vents was costly and as a result, they placed clothes in their windows and vents to keep mosquitoes from entering their houses. Other participants stated that they had many rooms in their houses hence it was costly to buy insecticides and spray all the rooms. It was also reported that as bricklaying was a major economic activity of the community, there were large pools of stagnant water in the area that served as breeding grounds for mosquitoes yet larviciding was expensive.

"*You may find that someone has got a six-bedroom house which will be costly to spray all the rooms with insecticides at once. Also, in a village like this one, you find that next to some households, are bricklaying sites which leave behind large pools of stagnant water, so larviciding here would be required but it is also expensive."* Male participant 5, FGD 7

Concerns were also raised about the potential side effects of certain malaria prevention methods on health therefore participants were reluctant to use them. Some participants mentioned that IRS, insecticide spraying and mosquito coils produced fumes that may affect the health of household members. As such, some community members did not desire to utilise these methods for fear of being harmful to their health including children.

"*I would have loved to use a mosquito coil but I am not comfortable with the scent it produces once it has been lit in the house, which is a related concern regarding the insecticide sprays as they may affect our breathing especially because of the fumes they produce."* Youth participant 2, FGD 10

Lack of knowledge about some malaria prevention approaches was identified as a limitation that prevented community members from using them. For example, some participants indicated that they had never heard of mosquito repellents neither did they know where to obtain them. As a result, they had difficulty using such unknown practices and instead used familiar malaria prevention techniques such as ITNs and destroying mosquito breeding sites. Similarly, some participants expressed concern that they did not know where to acquire IRS services, and lacked information on its potentially harmful effects on human health which hindered its use.

"*About the use of the IRS, we don’t know how to get it done, we don’t have anywhere to get the service in our area. Can IRS be harmful to an individual? At what time should spraying be done in the house and how would one manage to cover all the things in the house such that you don’t spray them? We don’t have answers to many of those questions."* Male participant 7, FGD 6

Another issue raised by participants was the inappropriate use of certain malaria prevention methods. From the KIIs, it was established that some community members did not use the ITNs given to them. A few individuals who used nets did not hang them properly which allowed mosquitoes to bite them whilst asleep. In addition, some community members used ITNs for undesignated roles such as fishing as well as barriers during poultry rearing and covering nursery beds for plants which were favoured due to being income-generating activities.

"*We have been part of this study of evaluating the government programme on the distribution of mosquito nets all over Uganda and we have witnessed that even after nets are distributed, a month later, either people are not using them or they are not hanging them the right way or you’ll find people using them for fishing or putting them to use for other purposes. So someone will say, ‘all my plants need to be covered in the nursery bed’ and instead of using the mosquito bed net for their protection, they will just take that same net and put it to other use that they consider more important to them at that point."* Malaria Researcher 2

In addition, the housing structure in the community, particularly the design of the windows, made it difficult to implement certain malaria prevention methods such as the installation of screens in windows and vents as noted by some key informants. In addition, due to poor physical planning for small-sized plots in urban and peri-urban settings, it was mentioned that some community members lacked enough space to dig drainage channels which led to stagnation of water around houses hence the breeding of mosquitoes.

"*If there was poor physical planning on small-sized plots, in a way it would facilitate mosquito breeding. For example, if a house is built on most of the plot area and there is no space for proper drainage, this water will just stagnate leading to mosquito breeding."* Wakiso District Health Team member

### Perspectives on the proposed RCT

From the FGDs, participants expressed their willingness to participate in the RCT on integrated malaria prevention. Some community members were willing to take part in the RCT because they wanted to better understand the use of the integrated approach to prevent malaria. This understanding would enable them to implement several malaria prevention methods in their households as well as raise awareness on the use of the integrated approach among other members of the community. As such, participants said it would prevent malaria and promote health in the area, as well as save money that would have been spent on medical expenses for malaria treatment.

"*Yes, we would give in our time and participate in the RCT so that we can gain more knowledge about the multiple methods of malaria prevention in our households so that we can also go back to our communities and teach the rest of the members about these methods."* Male participant 1, FGD 8

A variety of views and opinions were expressed by the key informants on the RCT on integrated malaria prevention. There were positive views from the key informants regarding the training of community members on integrated malaria prevention as part of the RCT. For instance, majority of the key informants noted that since the study would involve several households, community members involved would gain knowledge and also sensitise others hence increasing awareness on the use of multiple malaria prevention methods holistically. The key informants also emphasised that some community members had poor perceptions regarding some malaria prevention methods such as insecticide spraying which they believed might be harmful to their health. Therefore, sensitising community members about malaria prevention methods as part of the RCT would contribute to increasing community awareness on the use of non-conventional methods.

"*Being a large scale study, it will help that those people who are trained will go back and sensitise others. For instance, if they are 10 people trained and they also sensitise 10 more others and those 10 sensitise other 10 more people, then the message regarding malaria prevention would have spread among a large portion of the community." *CHW, Ngongolo parish

The proposed RCT was also noted to potentially have other benefits beyond malaria prevention. For instance, some key informants mentioned that malaria prevention methods such as maintaining sanitation through draining stagnant water and clearing overgrown vegetation would also prevent diarrhoeal diseases as well as other vectors and vermin such as snakes that might be harmful to people.

"*Keeping good sanitation at home is not only good for preventing malaria but also other diseases as well such as diarrhoea and so many other illnesses that one can get through poor conditions."* Health practitioner, Kajjansi Health Centre IV

From the key informants, it was reported that the use of multiple malaria prevention methods holistically meant that community members involved in the RCT would be exposed to a variety of methods from which to choose approaches that they preferred to implement in their households. In addition, the key informants noted that having a wide range of methods meant that people who may not use some methods such as ITNs because of reasons such as skin irritation, would be able to implement other methods such as closing their doors and windows early in the evenings or installing screening in windows and vents to prevent the entry of mosquitoes.

"*The proposed study is very good because it helps each person to choose for themselves the preferred method they can use to prevent malaria. For instance, one can choose to use an ITN or insecticide spraying if it happens to be the easier method for them to implement in the prevention of malaria."* CHW, Ngongolo parish

### Sustainability of integrated malaria prevention

There were various opinions provided by the participants regarding the sustainability of the interventions in the proposed RCT. From the key informants, it was mentioned that CHWs needed to be involved in the proposed RCT as this would then ensure sustainability of the malaria prevention methods in the community. It emerged that if CHWs were involved, they would continue with their roles of health education and community visits to encourage community members to implement malaria prevention methods holistically even after completion of the study. In addition, the FGD participants reported that they would be keen to continue with the use of multiple methods in malaria prevention beyond the RCT to continue preventing malaria in their settings.

"*Involving CHWs in the study would be crucial because even though the project would have ended, they will stay behind. CHWs can work together to make sure that the community continues implementing the various malaria prevention methods. Therefore, they would remain performing their routine roles in their communities concerning health promotion for malaria prevention."* CHW, Kitende parish

Furthermore, it was established that since some of the malaria prevention methods did not require much money to implement, they would be easy to sustain in the community. For instance, from the FGDs, some community members reported that they would continue with the implementation of malaria prevention methods such as clearing overgrown vegetation around their house, and draining stagnant water which do not require money to implement.

"*I would continue maintaining good sanitation, clearing overgrown vegetation and draining away stagnant water from my home as we wait for support regarding the other malaria prevention methods."* Female participant 6, FGD 3

However, it emerged that malaria prevention methods that are costly to implement would be harder to sustain by community members. For instance, from the FGDs, community members reported that they would only implement IRS and larviciding during the RCT hence may not implement these methods beyond the study since they were expensive. The key informants added that since most community members in rural settings were low-income earners, it would be difficult for them to buy larvicides to use in large water pools of water as these were expensive. As such, the key informants emphasised that malaria prevention methods such as larviciding would only be sustainable if community members acquired continuous support from the government or elsewhere.

"*There are some methods that we cannot continue implementing after the study because they are expensive, and we cannot afford them for instance larviciding in large pools of stagnant water. Additionally, despite the effect of IRS lasting for six months, I would not afford to continue using this method beyond the study unless I receive external support."* Female participant 7, FGD 7

## Discussion

Our study explored the feasibility of an RCT on integrated malaria prevention in rural communities in Uganda. Five major themes emerged from the study reflecting community and stakeholder perspectives concerning the integrated approach to preventing malaria. These themes were as follows: malaria prevention practices related to integrated malaria prevention; preferred malaria prevention methods in the integrated approach; potential challenges of integrated malaria prevention; perspectives on the proposed RCT and sustainability of integrated malaria prevention. This study builds on our previous work that explored experiences [1, 2], knowledge, practices and perceptions [1, 3–5], as well as the impact [6] of using integrated malaria prevention, some of which recommended conducting an RCT on the subject in future [2]. This increasing evidence on the integrated approach will not only inform our future research but also be used by other investigators interested in the use of multiple methods to prevent malaria at households and in the community. Such evidence on strategies to prevent malaria beyond ITNs and IRS is needed as part of national and global efforts to control the disease [27]. Indeed, the Uganda Malaria Reduction and Elimination Strategic Plan 2021-2025 recommends the use of a mix of interventions for various contexts to control the disease [28].

From the study, ITNs remain the major method of malaria prevention similar to other studies in the country [29–32]. This finding is not surprising considering government efforts to promote the method through routine distribution of LLINs to the populace for many years [30–33]. The negative effects reported with the use of nets included skin irritations which could be due to non-observance of instructions before their use such as not hanging the treated nets outdoors for 24 h before usage as recommended by the MOH [33]. Skin-related symptoms such as skin eruption, pruritus and paraesthesia have been noted as side effects from the chemicals used in the treatment of ITNs but these usually last a short period of time [34]. The practice of having the net outdoors reduces such skin effects that could emerge from their use. Misuse of nets such as for fishing and poultry rearing by some community members was noted in our study as well as other studies in Uganda and elsewhere [29, 35] which reduces the effectiveness of the method. Dealing with side effects and misuse of nets requires constant sensitisation and providing information to the community regarding good ITN practice and benefits. An interesting finding from our study was the use of plant repellents in the prevention of malaria, also reported by previous studies in Uganda [36, 37] and elsewhere [38]. Plant repellents for malaria prevention expand the list of choices for integrated malaria prevention hence need further research to support their enhanced use within the community.

A major finding from this study was the high willingness among community members to participate in the RCT which is promising. In our earlier research, we established that community members appreciated the integrated approach [3, 6, 7] and it led to an improvement in knowledge and related practices as well as reduction of malaria morbidity and mortality [1, 7]. Other studies elsewhere have also shown a high willingness of community members to support malaria control interventions beyond ITNs and IRS [39, 40]. Previous concerns and challenges in the use of several malaria prevention interventions holistically have been related to high costs, methods being many hence time consuming, limited knowledge on some methods and potential negative health effects [22, 40, 41]. Similar issues were highlighted in our study which require careful consideration in the design and implementation of the RCT especially as they would influence adoption of practices. The RCT would also need to provide sufficient information regarding the various methods in the integrated approach to fill the available knowledge gap that could affect practices. There is also a need to carefully consider the number of methods to be included in the RCT taking into consideration the community preferences and barriers.

Our study participants emphasised the need for sustainability of the interventions beyond the proposed RCT. Sustainability measures suggested included the use of CHWs during implementation which provides a great opportunity to ensure continuity of services beyond the study. Indeed, CHWs play a critical role in malaria control in many LMICs providing education to their communities, mobilising communities to take up interventions and treatment of children under 5 years [42, 43]. Therefore, prior involvement of CHWs in malaria control in the area suits their involvement in the RCT including community mobilisation, uptake of interventions and continued use of various malaria prevention methods after the study. CHWs have supported several community-based interventions for malaria control in many countries particularly in sub-Saharan Africa [44] which experiences could inform the RCT. Use of available resources in the community in the design of the RCT including CHWs is likely to lead to positive outcomes. It is also worth noting that sustainability of interventions in the integrated approach will also be determined by the methods promoted in the RCT as established in the study. Indeed, evidence suggests that malaria prevention methods that are costly such as larviciding are likely not to be used by communities without any external support [45].

A strength of this study is that it contributes important information regarding feasibility of the RCT on integrated malaria prevention which builds on the growing literature on integrated vector management. In addition, holding separate FGDs for women, men and youth ensured the various groups provided detailed information which could have been different if they had been combined. However, due to the COVID-19 pandemic restrictions in Uganda during data collection, some of the interviews were conducted remotely which rendered some interactions shorter as well as limited observance of non-verbal communication.

## Conclusions

There was high willingness among community members to participate in the proposed RCT on integrated malaria prevention due to the desire to learn about various approaches to prevent the disease. However, there were limitations identified in using multiple approaches to prevent malaria including high cost, and perceived negative health effects from the insecticide-based methods. Careful consideration needs to be given whilst choosing the type and number of methods to be included in the RCT. Along with the interventions, sustainability mechanisms need to be embedded in the RCT to ensure that the malaria prevention methods promoted have long-term benefits to the communities.

## Data Availability

Data and materials of the study are available from the corresponding author on reasonable request.

## Abbreviations

CHW: Community health worker
FGD: Focus group discussion
ITN: Insecticide-treated mosquito net
IRS: Indoor residual spraying
KII: Key informant interview
LLIN: Long-lasting insecticidal net
LMIC: Low- and middle-income country
MOH: Ministry of Health
NGO: Non-governmental organisation
RCT: Randomised controlled trial
